# Assessing left ventricular systolic function in shock: evaluation of echocardiographic parameters in intensive care

**DOI:** 10.1186/cc10368

**Published:** 2011-08-16

**Authors:** Lill Bergenzaun, Petri Gudmundsson, Hans Öhlin, Joachim Düring, Anders Ersson, Lilian Ihrman, Ronnie Willenheimer, Michelle S Chew

**Affiliations:** 1 Department of Anaesthesiology, Institution of Clinical Sciences, Entrance 42, Skåne University Hospital, Lund University, Södra Förstadsgatan 101, S-20502 Malmö, Sweden; 2 Department of Biomedical Science, Malmö University, Södra Förstadsgatan 101, S- 20506 Malmö, Sweden; 3 Department of Cardiology, Institution of Clinical Sciences, Skåne University Hospital, Lund University, Getingevägen 4, S- 22185 Lund, Sweden; 4 Department of Intensive Care Medicine, Institution of Clinical Sciences, Entrance 42, Skåne University Hospital, Lund University, Södra Förstadsgatan 101, S-20502 Malmö, Sweden; 5 Heart Health Group, Lund University, Geijersg. 4C, 21618 Limhamn, Sweden

## Abstract

**Introduction:**

Assessing left ventricular (LV) systolic function in a rapid and reliable way can be challenging in the critically ill patient. The purpose of this study was to evaluate the feasibility and reliability of, as well as the association between, commonly used LV systolic parameters, by using serial transthoracic echocardiography (TTE).

**Methods:**

Fifty patients with shock and mechanical ventilation were included. TTE examinations were performed daily for a total of 7 days. Methods used to assess LV systolic function were visually estimated, "eyeball" ejection fraction (EBEF), the Simpson single-plane method, mean atrioventricular plane displacement (AVPDm), septal tissue velocity imaging (TDIs), and velocity time integral in the left ventricular outflow tract (VTI).

**Results:**

EBEF, AVPDm, TDIs, VTI, and the Simpson were obtained in 100%, 100%, 99%, 95% and 93%, respectively, of all possible examinations. The correlations between the Simpson and EBEF showed *r *values for all 7 days ranging from 0.79 to 0.95 (*P *< 0.01). the Simpson correlations with the other LV parameters showed substantial variation over time, with the poorest results seen for TDIs and AVPDm. The repeatability was best for VTI (interobserver coefficient of variation (CV) 4.8%, and intraobserver CV, 3.1%), and AVPDm (5.3% and 4.4%, respectively), and worst for the Simpson method (8.2% and 10.6%, respectively).

**Conclusions:**

EBEF and AVPDm provided the best, and Simpson, the worst feasibility when assessing LV systolic function in a population of mechanically ventilated, hemodynamically unstable patients. Additionally, the Simpson showed the poorest repeatability. We suggest that EBEF can be used instead of single-plane Simpson when assessing LV ejection fraction in this category of patients. TDIs and AVPDm, as markers of longitudinal function of the LV, are not interchangeable with LV ejection fraction.

## Introduction

Echocardiography is a useful tool for assessing cardiac function in hemodynamically unstable patients in the intensive care unit (ICU) [1]. For example, impairments of both systolic and diastolic left ventricular (LV) function have been observed in sepsis [2,3]. Only a few longitudinal transthoracic echocardiographic (TTE) [4-6] studies have been made of the LV systolic function in ICU patients with shock. Previous investigators have mainly concentrated on either acute changes [3,7,8] or used the transesophageal approach [9,10]. Serial evaluation of LV systolic function by echocardiography may provide additional insight into the relation between cardiac function and critical illness. A number of methods can be used to assess LV systolic function with echocardiography. These are well described in patients with cardiac disease and in healthy subjects, but few data exist from ICU patients. The different parameters do not necessarily assess the same features of LV systolic function. Their association to each other and how they change over time in the critically ill setting is unknown. LV ejection fraction (LVEF) can be assessed by the single- [11] or biplane [12] Simpson method, or more rapidly by visual estimation, "eyeball" ejection fraction (EBEF) [13,14]. Although earlier studies have shown that satisfactory images can be obtained in only about 70% of patients with cardiac disease [15], or even less in ventilated patients [16], the development of second harmonic imaging [17] has improved endocardial border delineation, and the technique is today a standard feature in most modern ultrasound devices. Calculation of stroke volume (SV) in the LV outflow tract (LVOT) requires measurements of the velocity time integral (VTI) in LVOT and LVOT cross-sectional area. The latter can be difficult to obtain and is a known source of error in cardiac output (CO) measurement [18,19]. LVOT VTI, also called stroke distance, is feasible and reproducible in patients with cardiac disease [20] and is an accepted measure of LV function under changing hemodynamic conditions in experimental settings [21,22]. In patients with cardiac disease, left atrioventricular plane displacement (AVPD), also called mitral annulus movement, is a valuable tool for the assessment of longitudinal LV systolic function and is fairly easy to obtain [23,24]. The systolic pulsed tissue Doppler velocity of the LV septal wall (TDIs) is another new index of the longitudinal LV systolic function [25,26]. Both these parameters have been validated against LVEF measured by different methods in patients with cardiac disease [27,28]. In critically ill patients, tissue Doppler has predominantly been used in the assessment of LV diastolic function [7,9] although recently also as a parameter for LV systolic function [8,29]. The aim of this study was to describe the feasibility, association between, and repeatability of, the different methods of evaluating LV systolic function in mechanically ventilated patients with shock, by using TTE.

## Materials and methods

The study was approved by the Regional Ethics Review Board, Lund, Sweden (Dnr.187/2005). Informed consent was sought from the patient or, if not possible, from the next of kin. The study design was a single-center prospective observational cohort study of critically ill patients. Patients admitted to the mixed-bed ICU of Malmö University Hospital, Sweden, were screened for eligibility, and we included 55 consecutive patients with shock, defined as failure to maintain mean arterial pressure ≥70 mm Hg despite adequate fluid resuscitation according to the surviving sepsis campaign algorithm [30]. All patients were older than 18 years, and all patients were mechanically ventilated. Exclusion criteria were pregnancy, known abnormalities of coagulation, fibrinolytic therapy, compromised immunity, or a "Do Not Attempt Resuscitation" order. Patients could be included only once. The study period was 7 days but was shorter in case of discharge from the ICU or death. Acute Physiology and Chronic Health Evaluation (APACHE) II scores [31] were calculated at admission, and Sequential Organ Failure Assessment (SOFA) scores [32] were calculated for each day of the study period. After the initial resuscitation period, fluids were given at the treating clinician's discretion.

### Transthoracic echocardiography

TTE examinations were performed within 12 hours of inclusion into the study. Subsequent studies were conducted daily for 7 days or until death or discharge from the ICU. The examinations were performed by either of four experienced echocardiographers (LB, MC, PG, MD). Images were acquired by using a Hewlett-Packard Sonos 5500 (Andover, MA, U.S.A.) scanner and a 3-MHz transthoracic transducer. Two-dimensional (2D) imaging examinations were performed in the standard apical four- and two-chamber views. Tissue harmonic imaging was used to enhance 2D image quality. These images were used to estimate LVEF by the EBEF and modified single-plane Simpson method. M-mode images were obtained at the LV septal, lateral, anterior, and posterior borders of the mitral ring [33] in the apical four- and two-chamber views, and an average AVPD value was calculated from these four locations (AVPDm). Pulsed-wave tissue Doppler recorded the peak systolic velocity (TDIs) of the LV septal wall at the level of the mitral annulus in the apical four-chamber view. The VTI of the LVOT was measured with pulsed wave Doppler in the apical five-chamber view. All TTE studies were recorded and measurements were taken in triplicate and averaged. Analysis of the measurements was made in Phillip's digital storing program Xcelera (Best, the Netherlands) offline. All analyses were made by one observer (LB). For repeatability studies, 15 randomly selected patients were re-analyzed after a two-year interval to determine intraobserver repeatability (LB). In addition, another highly experienced investigator (PG) independently analyzed the images to determine interobserver repeatability.

### Statistical analysis

Data are presented as median (lower quartile: upper quartile). Normality was tested for using the Kolmogorov-Smirnov test. For temporal changes, a repeated-measure ANOVA (Kruskall-Wallis test for nonparametric data) was applied. For correlation between two variables, the Spearman rank correlation was used, and for differences between two groups, the Mann-Whitney *U *test was used. For comparison of paired values, a Wilcoxon signed-rank test was used, with a Bonferroni correction applied for multiple analyses. The intra- and interobserver variability was measured by the coefficient of variation (CV). CV was defined as the ratio of the standard variation to the mean multiplied by 100. Significance was set at *P *< 0.05. The analyses were performed by using SPSS 17.0 for Windows.

## Results

The original study included 55 consecutive patients. Two patients were excluded because of lack of written consent. One patient died 4 hours after study inclusion and before echocardiographic examination; one patient was morbidly obese and TTE was not possible; and one patient was incorrectly registered in the echocardiography database. These five patients were excluded from statistical analysis. Of 350 expected echocardiographic examinations, 91 were missing because of death or discharge from the ICU before Day 7 (25 of 50 patients). Another 28 examinations were lost during the installation of a new offline storage and analysis system. Thus, in total, 231 examinations were available for analysis. Patient characteristics are shown in Table 1. Two thirds of the population had septic shock. The remaining patients had shock due to other causes (pancreatitis, post-major noncardiac surgery, intoxication and multiorgan failure, gastrointestinal bleeding and portal hypertension, or unknown cause). Mechanical ventilations occurred in 45 (90%) patients at inclusion and in all patients (100%) during the first 2 days of the study period. Of 231 echocardiographic examinations, 227 (98%) were performed with the patient mechanically ventilated. Pre-existing cardiac disease was present in 24% of patients, defined as severe arrhythmia, heart failure, or ischemic heart disease. In all, 48% had pre-existing treatment with β-blockers, ACE-inhibitors, Ca-channel blockers, and/or nitrates. All patients were receiving vasopressors at inclusion.

**Table 1:** Patient characteristics

Characteristic	Values
Number of patients	50
Gender (M/F)	36/14
Age (years)	65 (54:74)
SOFA day 1	12 (9:14)
APACHE II	24 (19:29)
ICU LOS	8 (4:13)
Days on mechanical ventilation	6.5 (3:13)
Vasopressor μg/kg/min	0.09 (0.05: 0.14)
Cardiac disease (%)	24
Preexisting therapy (%)	48
ICU mortality (%)	24

Data are presented as median (lower quartile:upper quartile). APACHE, Acute Physiology and Chronic Health Evaluation; ICU, intensive care unit; LOS, length of stay; SOFA, Sequential Organ Failure Assessment. Norepinephrine was used as vasopressor. Twelve patients received dobutamine, and one, adrenaline at inclusion. Ten patients received levosimendan during the study period.

### Assessment of feasibility

Both EBEF and AVPDm could be assessed in 100% of the investigations. TDIs and LVOT VTI could be assessed in 99% and 95%, respectively. In 7% of the investigations, the Simpson single-plane method could not be used to evaluate LVEF, because of poor image quality. On average, LV systolic function improved significantly over the 7-day observation period, as did SOFA score (*P *< 0.05). All measured LV systolic function parameters were significantly improved on day 6 compared with day 1 (Table 2). LV systolic function measurements on day 1 did not differ significantly between those who survived the 7-day study period and those who did not. Highly significant correlations were seen between the Simpson method and EBEF for all days (*r *= 0.794 to 0.949; *P *< 0.01). AVPDm, TDIs, and LVOT VTI correlated with the Simpson method, although these correlations were weaker than those seen between Simpson and EBEF (Table 3). The Simpson method showed considerable variations in correlations with AVPDm, TDIs, and LVOT VTI over the 7-day period. There were similar results for AVPDm versus TDIs, in which the correlation was reasonable at day 1 (*r *= 0.427; *P *< 0.01) and on average for all measurements (*r *= 0.439; *P *< 0.01), but varied considerably between days (Table 3).

**Table 2:** Systolic left ventricular function from Day 1 to Day 7

	Day 1 *n *= 47	Day 2 *n *= 44	Day 3 *n *= 34	Day 4 *n *= 31	Day 5 *n *= 28	Day 6 *n *= 26	Day 7 *n *= 21	*p *value (over time)
Simpson (%)	50 (41:58)	53 (45:62)	53 (45:58)	56 (48:64)	59 (50:63)	62 (57:71)^a^	62 (54:65)	< 0.05
EBEF (%)	49 (40:55)	50 (40:65)	50 (40:59)	52 (45:63)	58 (45:61)^a^	65 (55:70)^a^	65 (50:70)^a^	< 0.05
AVPDm (mm)	10.5 (8.0:12.6)	11 (9.1:12.6)	11 (9.0:13.0)	12 (9.1:14.0)	11 (9.9:14.0)	12 (10.0:14.8)^a^	12.5 (11.0:14.0)^a^	< 0.05
TDIs (cm/sec)	8.9 (7.1:10.0)	9.1 (7.6:10.4)	8.3 (7.1:10.0)	9 (7.4:10.0)	9.3 (8.5:11.0)	10 (8.9:12.3)^a^	11 (9.0:13.5)	< 0.05
LVOT VTI (cm)	18 (15:23)	20 (17:26)^a^	21(18:24)^a^	21(18:24)^a^	22 (18:24)^a^	22 (20:24)^a^	23 (20:24)	< 0.05
SOFA	12 (9:14)	10 (8:13)^a^	8 (6:12)^a^	8 (6:11)^a^	7 (5:10)^a^	8 (5:9)^a^	7 (5:9)^a^	< 0.05

Simpson, the Simpson single-plane method of the four-chamber view; AVPDm, atrioventricular plane displacement, mean value of septal, lateral, anterior, inferior measurements; EBEF, eyeball ejection fraction; LVOT VTI, velocity time integral in the left ventricular outflow tract; SOFA, Sequential Organ Failure Assessment score; TDIs, pulsed tissue Doppler imaging, systolic velocity of the septal portion of the mitral annulus. Data are presented as median (lower quartile:upper quartile). *P *indicates statistical difference over time (Kruskall-Wallis). ^a^Statistical significant difference compared to Day 1 (Wilcoxon signed-rank test with Bonferroni correction)

**Table 3:** Correlations (*r*) of systolic parameters and level of significance (*P*)

	Day 1 *n *= 47		Day 2 *n *= 44		Day 3 *n *= 34		Day 4 *n *= 31		Day 5 *n *= 28		Day 6 *n *= 26		Day 7 *n *= 21		All measurements *n *= 253	
	***r***	***P***	***r***	***P***	***r***	***P***	***r***	***P***	***r***	***P***	***r***	***P***	***r***	***P***	***r***	***P***

Simpson vs. EBEF	0.896	^a^	0.949	^a^	0.910	^a^	0.859	^a^	0.855	^a^	0.794	^a^	0.942	^a^	0.905	^a^
Simpson vs. AVPDm	0.495	^a^	0.346	^b^	0.281		0.392	^b^	0.322		0.101		0.577	^a^	0.404	^a^
Simpson vs. TDIs	0.609	^a^	0.559	^a^	0.530	^a^	0.413	^b^	0.152		0.123		0.310		0.473	^a^
Simpson vs. LVOT VTI	0.616	^a^	0.408	^a^	0.288		0.499	^b^	0.570	^a^	0.450	^b^	0.742	^a^	0.513	^a^
AVPD vs. TDIs	0.427	^a^	0.264		0.456	^a^	0.356		0.535	^a^	0.635	^a^	0.630	^a^	0.439	^a^
^a^	Corr sign at the 0.01 level.															
^b^	Corr sign at the 0.05 level.															

Simpson single-plane method of the four-chamber view. AVPDm, atrioventricular plane displacement, mean value of septal, lateral, anterior, inferior measurements; EBEF, eyeball ejection fraction; LVOT VTI, velocity time integral in the left ventricular outflow tract; TDIs, pulsed tissue Doppler imaging, systolic velocity of the septal portion of the mitral annulus; Spearman rank correlation was used, and for differences between two groups, Mann-Whitney *U *test was used. *P *values have been corrected by the Bonferroni method.

### Repeatability of measurements

Intraobserver repeatability ranged from 3.1% to 10.6%, being worst for the Simpson single-plane method (Table 4). Similar findings were seen for interobserver repeatability (Table 4).

**Table 4:** Reproducibility of measurements

	EBEF	Simpson	TDIs	AVPDm	LVOT VTI
Intraobserver	6.8	10.6	8.2	4.4	3.1
Interobserver	9.9	8.2	7.2	5.3	4.8

Intra- and interobserver coefficients of variation (%) for EBEF; the Simpson single-plane method of the four-chamber view; AVPDm, atrioventricular plane displacement, mean value of septal, lateral, anterior, inferior measurements; EBEF, eyeball ejection fraction; LVOT VTI, velocity time integral in the left ventricular outflow tract; TDIs, pulsed tissue Doppler imaging, systolic velocity of the septal portion of the mitral annulus.

## Discussion

The main findings of this study area follow:

1. Daily measurements of LV systolic function with EBEF, Simpson, AVDPm, LVOT VTI, and TDIs were all feasible in a group of hemodynamically unstable patients.

2. Serial measurements revealed that all LV systolic parameters improved significantly over time.

3. Good correlations were observed between EBEF and the Simpson single-plane method throughout the entire observation period. In contrast, poorer correlations were seen for Simpson versus AVPDm and Simpson versus TDIs, which varied substantially during the observation period.

4. An acceptable repeatability was found, with best results seen for LVOT VTI and AVPDm.

### Feasibility

Our study demonstrated a good feasibility for all methods for the estimation of LV systolic function. Images were obtainable in 93% to 100%, with the EBEF and AVPDm methods providing best results (100% of examinations). This is somewhat surprising, given a commonly accepted view that TTE imaging is difficult in mechanically ventilated patients. However, our results are in agreement with recent studies, in which the feasibility of acceptable TTE images in ICU patients was as high as 97% to 99% [34,35], with the apical view offering the best window [35]. From a clinical point of view, the excellent feasibility demonstrated in the present study supports the use of routine TTE, even in very ill, mechanically ventilated patients. This is a clear advantage, considering that TTE is considerably less invasive than transesophageal echocardiography.

### Systolic function over time

Regardless of the method used, LV systolic function improved significantly over time, reflecting the clinical course of the patients. AVPDm and TDIs, as markers of the long-axis function of the LV, seemed to mirror clinical improvement, as did all other indices of LV systolic function. *Post hoc *analyses showed that all markers of LV systolic function were significantly improved on day 6 compared with day 1, reflecting the clinical observation that most (44 of 50) patients were weaned of vasopressors and inotropes by day 6.

### Correlations between the Simpson method and other methods

The biplane Simpson method is a widely recommended method for the evaluation of LVEF [12]. Quantification of LVEF by the single-plane Simpson method correlates strongly with biplane Simpson in postinfarction patients and reliably detects small changes in LVEF [11]. EBEF can be performed more rapidly and has previously been shown to correlate well with more-formal quantitative assessments of LVEF, such as the Simpson single-plane and biplane EF [14], radionucleotide EF [13], and biplane contrast ventriculography [36] in patients with cardiac disease. EBEF is also independent of endocardial border tracing, which can be difficult in suboptimal images, an important limitation in ventilated patients. EBEF also integrates information about global and regional contractility, which may have advantages in a setting in which variations in regional contractility may reduce the accuracy of Simpson. Our study is in agreement with earlier studies showing good correlation between Simpson single-plane method and EBEF. We have further shown that this correlation is consistent throughout the entire observation period (Days 1 to 7). In contrast to EBEF, correlations between Simpson and AVPDm, Simpson and TDIs, and even the correlations between AVPDm and TDIs varied substantially over the observation period. Previous studies in patients with cardiac disease have shown varying correlations between LVEF measured by radionucleotide or Simpson and AVPD or TDI, ranging from good [37,38] to poor [39,40]. In healthy subjects, the correlations were rather poor [26,38,41,42].

AVPD and TDI may be superior in detecting subtle abnormalities of LV systolic function [43] and in patients with subclinical heart disease [44,45], as well as with increasing age [46]; longitudinal contractility is reduced whereas LVEF is preserved. A number of possible mechanisms exist by which subendocardial, longitudinal LV function may become impaired earlier than subepicardial, circumferential LV function [47]. Our observations and several previous findings emphasize that neither AVPD [39,43,46] nor TDI [25] is interchangeable with LVEF, and by using LVEF solely when describing LV systolic function, the long-axis function of the heart is not necessarily considered. We found only moderate associations between AVPDm and TDIs, and they may also not be interchangeable, although TDI correlated well with AVPD in healthy individuals [26,48]. Several possible reasons may explain a lack of correlation between TDI and AVPD [23,49,50].

We showed that LVOT VTI was easily accessible and had a good intra- and interobserver repeatability. This is in line with previous studies in patients with aortic valve stenosis [51] and in other patients with cardiac disease [20]. Of note, animal studies have shown that LVOT VTI is an effective and noninvasive method when assessing LV systolic function under conditions of varying preload, heart rate, and inotropic state [21,22,52], an important asset in hemodynamically unstable patients. LVOT VTI is thought to be a better indicator of LV systolic function than is stroke volume, without the confounding factor of LVOT-area measurements [18,19,51]. This is especially true for ICU patients for whom usable images in the transthoracic parasternal view (used to measure LVOT diameter) can be obtained in about two thirds of patients [34].

### Repeatability

The Simpson method showed the worst repeatability, which is comparable with the results of Lamia *et al. *in critically ill patients [53]. The repeatability using the EB technique was comparable. In contrast, we found good intra- and interobserver repeatabilities for LVOT VTI measurements. For the measurements of longitudinal LV systolic motion, we found clinically acceptable repeatability. We believe that these findings are clinically relevant, because the Simpson is the commonly recommended method for LV systolic function assessment, yet provided the poorest repeatability in this study.

### Limitations

The influence of vasopressors, inotropes, and mechanical ventilation on echocardiographic measurements is uncertain. We have not excluded patients with previous known heart failure or atrial fibrillation, nor have we excluded patients with onset of atrial fibrillation during their critical illness, which might have influenced our results. Our intention was to investigate echocardiographic parameters that are well established in patients with cardiac disease, but less investigated in critically ill, mechanically ventilated patients with shock. We could have used contrast agents to facilitate the endocardial border detection, but this was not advisable at the time of data collection [54]. The strength of this study is the temporal approach and the simultaneous comparisons of five different, commonly used methods for the evaluation of LV systolic function. We found good feasibility and clinically acceptable reproducibility for our measurements, but all studies were conducted by experienced operators only. Echocardiography is user dependent, and results may be poorer in less-experienced hands.

## Conclusions

Measurement of LV systolic function was feasible in hemodynamically unstable, mechanically ventilated patients. The different methods for the assessment of LV systolic function all reflected improvement in clinical state over time. However, the different methods did not all correlate well to one another, probably because they measure different components of LV systolic function and presumably do not change uniformly over time. EBEF and AVPDm provided the best, and Simpson, the worst feasibility. Additionally, Simpson showed the poorest repeatability. We suggest that EBEF can be used instead of single-plane Simpson when assessing LVEF and that combining different left ventricular indices, like EBEF and AVPD, when measuring LV systolic function, might be valuable in this category of patients.

## Key messages

• Assessment of left ventricular (LV) systolic function by transthoracic echocardiography (TTE) using five different echocardiographic parameters over a 7-day period in ventilated patients with shock.

• All parameters were easy obtainable (93% to 100%) and had acceptable repeatability (coefficient of variation, 3.1% to 10.6%); their correlation to one another showed substantial variation (*r *= 0.101 to 0.949) over time.

• Eyeball ejection fraction (EBEF) can be used instead of single-plane Simpson when assessing LV ejection fraction, but markers of the LV longitudinal function, such as tissue Doppler and atrioventricular plane displacement, are not interchangeable with LV ejection fraction.

## Abbreviations

AVPDm: atrioventricular plane displacement mean; CO: cardiac output; CV: coefficient of variation; EBEF: eyeball ejection fraction; EF: ejection fraction; ICU: intensive care unit; LV: left ventricular; LVOT: left ventricular outflow tract; TDIs: tissue Doppler imaging septal; TTE: transthoracic echocardiography; VTI: velocity time imaging.

## Competing interests

The authors declare that they have no competing interests.

## Authors' contributions

LB made substantial contributions to the conception and design of the study, participated in interpretation of data, made substantial contributions in acquisition and analysis of data, and helped to draft the manuscript. RW and AE made substantial contributions to conception and design of the study. MC made substantial contributions to conception and design of the study, participated in interpretation of data, and helped to draft the manuscript. PG made substantial contributions to conception and design of the study and made substantial contributions to acquisition and analysis of data. JD made substantial contributions to conception and design of the study and made contributions to the acquisition of data. HO participated in interpretation of data and helped to draft the manuscript. LI made contributions to the acquisition of data. All authors have made substantial intellectual contributions to the manuscript and have given final approval of the version to be published.
